# Sensitivity-Compensated Micro-Pressure Flexible Sensor for Aerospace Vehicle

**DOI:** 10.3390/s19010072

**Published:** 2018-12-25

**Authors:** Xiaozhou Lü, Jianan Jiang, Hui Wang, Qiaobo Gao, Shaobo Zhao, Ning Li, Jiayi Yang, Songlin Wang, Weimin Bao, Renjie Chen

**Affiliations:** 1 The School of Aerospace Science and Technology, Xidian University, Xi’an 710071, China; jiangjianan4213@126.com (J.J.); whui94@126.com (H.W.); 15991262350@sina.cn (Q.G.); imnazi@163.com (S.Z.); lining408060@163.com (N.L.); yang.jiayi@stu.xidian.edu.cn (J.Y.); slwang@mail.xidian.edu.cn (S.W.); baoweimin@cashq.ac.cn (W.B.); 2 Beijing Key Laboratory of Environmental Science and Engineering, School of Chemical Engineering and the Environment, Beijing Institute of Technology, Beijing 100081, China

**Keywords:** aerospace, flexible sensor, micro-pressure measurement, sensitivity compensation

## Abstract

When flight vehicles (e.g., aerospace vehicles, Low Earth Orbit (LEO) satellites, near-space aircrafts, Unmanned Aerial Vehicles (UAVs) and drones) fly at high speed, their surfaces suffer the micro-pressure from high-altitude thin air. The long-term effect of this pressure causes the surface components of flight vehicle to deform or fall off, which can lead to a serious accident. To solve this problem, this paper proposes a sensitivity-compensated micro-pressure flexible sensor based on hyper-elastic plastic material and plate parallel capacitance. The sensor is able to measure a range of 0–6 kPa micro-pressure suffered by the flight vehicle’s surface with high sensitivity and flexible devices. In this paper, we propose the principle, structure design and fabrication of the sensitivity-compensated micro-pressure flexible sensor. We carried out experiments to obtain the static characteristic curve between micro-pressure and the output capacitance of the sensor devices, and investigated the relationship between sensitivity and geometric parameters. We also compared the performance of the flexible sensor before and after sensitivity compensation. The result shows that the sensor can measure a range of 0–2 kPa and 2–6 kPa with a sensitivity of 0.27 kPa^−1^ and 0.021 kPa^−1^, which are 80% and 141.38% higher than the sensor before compensation; a linearity of 1.39% and 2.88%, which are 51.7% and 13.1% higher than the sensor before compensation; and a hysteresis and repeatability of 4.95% and 2.38%, respectively. The sensor has potential applications in flight vehicles to measure the micro-pressure with high sensitivity and flexibility.

## 1. Introduction

Flight vehicles are considered as one of the key flight equipment types in the 21st century since they can fly at ultra-high-speed in the near space and have the functions of aircraft, carriers and spacecraft, etc. [1]. The surface of flight vehicles (such as aerospace vehicle, Low Earth Orbit (LEO) satellites, near-space aircrafts, Unmanned Aerial Vehicles (UAVs) and drones) suffer the micro-pressure from high-altitude thin air when they are flying at high speed. Long-term effect of this pressure can deform the surface components, which could lead to a serious accident [2]. Therefore, it is important to develop sensor to measure this kind of micro-pressure.

An aerospace vehicle primarily flies in the atmosphere of the troposphere, the stratosphere and the mesosphere (especially the stratosphere and the mesosphere) at the height of about 20–70 km [3]. The height where spacecraft is located still exists the impact of thin atmosphere and harsh ambient temperature. It can lead to components wearing out or even being damaged after many hours of service.

When the spacecraft is static in the stratosphere, the ambient temperature is approximately from −53.2 °C to 0 °C and the flying height range is about 20–50 km. Pressure (P) equals 5.46 kPa at the height of 20 km, and the pressure is close to 0.5 kPa at the height of 50 km. When the aerospace vehicle is in the mesosphere, the ambient temperature changes from −55 °C to 0 °C and the altitude is about 50–70 km. The air at this altitude is very thin. According to the atmospheric temperature vertical profile [3], the maximum pressure reaches 0.5 kPa and the lowest is 0.05 kPa.

An aerospace vehicle has a high speed when flying dynamically. The effect of the thin air in near space will generate a dynamic resistance $F_{1}$ [4], which can be calculated by

(1) $$F_{1}=\frac{1}{2}\cdot \rho \cdot C_{D}\cdot v^{2}\cdot A$$

where $C_{D}$ is the air resistance coefficient, v is the velocity of the object relative to the fluid, A is the windward area of the object, and ρ is the air density at the current altitude (the $\rho_{0}$ of normal dry air is 1.293 kg/m^3^). The air density at the current altitude ρ can be calculated by

(2) $$\rho =\frac{P\cdot T_{0}\cdot \rho_{0}}{P_{0}\cdot T}$$

where P is the current altitude pressure, $P_{0}$ is the standard atmospheric pressure, $T_{0}$ is the Kelvin temperature at zero degrees, and T is the current altitude Kelvin temperature. The air resistance coefficient $C_{D}$ of the spacecraft is $0.08^{-1}$ and the velocity v, which is less than that in airplane because of the thinner air in near space, is 200–700 km/h. Substituting the parameters $C_{D}$ and v into Equations (1) and (2), the result is about 0.165 kPa.

In summary, according to analysis of the pressure distribution, temperature change and flight resistance of the aerospace vehicle, we obtain the result that the pressure range is 0–6 kPa and the minimum temperature is −55 °C when an aerospace vehicle flying. The pressure sensor for aerospace vehicle should detect a range of 0–6 kPa micro-pressure at an extreme temperature of −55 °C.

Many pressure sensors have been developed and applied in the process of spacing pressure measurement. In principle, these sensors can be divided into the following types: resistance [5,6,7,8,9], piezoelectric [10,11], magnetic [12], fiber optic [13,14,15,16], resonant [17] and capacitive [18,19,20].

Resistance sensors measure micro-pressure according to piezoresistive effect of pressure sensitive materials. Liwei Lin et al. [5] designed a MEMS pressure sensor based on piezoresistive sensing principle. The sensor has a thickness of 2 µm, 100 µm width and a measurement range of 100 Pa with a maximum linearity error of ±1%. This sensor has been applied in aerospace for micro-pressure measurement but it is rigid. Jianli Cui et al. [6] and Yin He et al. [7] presented flexible pressure sensors based on CNTs-PDMS composite and m-MWNTs-PU films, respectively. These sensors are flexible but still have challenge in sensitive for micro-pressure measurement. Huang et al. [8] produced a piezoresistive micro-pressure sensor using conductive carbon black/graphene and polymer sponge with a high sensitivity of 0.38 kPa^−1^ at 0–1 kPa. Y Jung et al. [9] proposed a piezoresistive sensor based on flexible core and four elastomers. The sensor has a high-sensitivity of 0.165 kPa^−1^ in the shear direction and 0.0173 kPa^−1^ in the normal direction. However, these sensors still have difficulty measuring micro-pressure for aerospace in a harsh environment (low atmospheric pressure, and high and low temperature).

Piezoelectric sensors measure micro-pressure according to piezoelectric effect of piezoelectric materials. Yu-Jen Hsu et al. [10] and Ping Yu et al. [11] presented flexible piezoelectric pressure sensors based on PVDF. These sensors have a high sensitivity and good flexibility but bad static characteristics of linearity and measurement range.

Many other types of sensors (magnetic [12], fiber optic [13,14,15,16] and resonant [17], etc.) have also been presented for micro-pressure measurement. However, these sensor still have challenges of flexibility and miniaturization.

Capacitance sensors [18,19,20], which have the advantages of temperature insensitivity and micro size, have been applied to measure micro-pressure. X. Lu et al. [18] presented a three interfacial stress capacitance sensor for planta and prosthesis pressure measurement. Lixin Chen et al. [19] presented a 4×4 capacitive pressure tactile sensor fabricated with new materials. Shigeru Toyama et al. [20] also presented a sheet-type shear force sensor and a measurement system based on liquid electrolyte and elastic rubber ring. However, few works discuss the possibility to aerospace vehicle application due to complicated environment (low atmospheric pressure, high and low temperature, etc.) and strict requirements (flexible, simple structure, highly sensitive, simple fabrication, reliable, etc.).

Aiming at this problem, this paper presents a sensitivity-compensated micro-pressure flexible sensor based on plate parallel capacitance and hyper-elastic plastic material for aerospace vehicle application. We propose the principle of sensitivity-compensated micro-pressure flexible sensor based on parallel plate capacitance. We present the structure design and fabrication of the sensor. We investigated the flight environment of high speed aircraft. We present the application of the sensor in this environment. We conducted experiments to obtain the static characteristic curve between micro-pressure and output capacitance. We also compared the performance of the sensor before and after sensitivity compensation to verify the effectiveness of sensitivity compensation. The conclusion is presented at the end. By using this sensor structure, our sensor has higher sensitivity (in the range of 0–2 kPa and 2–6 kPa with a sensitivity of 0.27 kPa^−1^ and 0.021 kPa^−1^), higher linearity (1.39% and 2.88%), and has potential application in aerospace vehicles, LEO satellites, near-space aircrafts, Unmanned Aerial vehicles (UAVs) and drones to measure the micro-pressure caused by high-altitude thin air.

## 2. Methods

### 2.1. The Measurement Principle of the Flexible Micro-Pressure Sensor

The micro-pressure sensor for aerospace vehicles presented in this work is based on the principle of parallel plate capacitance. The capacitance of parallel plate capacitor without considering the edge effect is $\frac{\varepsilon_{0}\varepsilon_{r}\cdot A}{d}$, where A is the overlap area between the plates, d is the spacing between the parallel plates, $\varepsilon_{0}$ is dielectric constant of the vacuum and $\varepsilon_{r}$ is the relative dielectric permittivity of the dielectric layer.

Supposing the initial spacing between the plate of the sensor is a constant, the initial value of the capacitor is $C_{0}$. If the spacing between the plates changes, ∆d, the capacitance value (C) will be

(3) $$C=C_{0}+∆C=\frac{\varepsilon A}{d_{0}-∆d}=\frac{\varepsilon A(1+\frac{∆d}{d_{0}})}{d_{0}\left(1-{\left(\frac{∆d}{d_{0}}\right)}^{2}\right)}$$

When $∆d\ll d_{0}$, that is, the range is much smaller than the initial distance between the plates, Equation (1) can be simplified as

(4) $$C=\frac{\varepsilon A(1+\frac{∆d}{d_{0}})}{d_{0}}=C_{0}+C_{0}\cdot \frac{∆d}{d_{0}}$$

According to the elastic mechanics, the Young’s modulus E of an elastic material can be calculated by

(5) $$E=\frac{\sigma }{\varepsilon_{d}}=\frac{F/A}{∆d/d_{0}}$$

where σ is the stress and $\frac{F}{A}$ means the force per unit area. $\varepsilon_{d}$ is the strain and $\frac{∆d}{d_{0}}$ means relative deformation under external force. Equation (5) can also be written as

(6) $$\frac{∆d}{d_{0}}=\frac{F/A}{E}$$

Substituting Equation (6) into Equation (4), we can obtain:

(7) $$C=C_{0}+C_{0}\cdot \frac{∆d}{d_{0}}=\frac{\varepsilon A}{d_{0}}\left(1+\frac{F}{AE}\right)=\frac{\varepsilon }{Ed_{0}}F+\frac{\varepsilon A}{d_{0}}$$

The relative change in capacitance $\left(\frac{∆C}{C_{0}}\right)$ is now

(8) $$\frac{∆C}{C_{0}}=\frac{\frac{∆d}{d_{0}}}{1-\frac{∆d}{d_{0}}}$$

The sensitivity (*S*) of the measuring method can be obtained as

(9) $$S=\frac{\frac{∆C}{C_{0}}}{F}=\frac{∆d}{d_{0}-∆d}\cdot \frac{1}{F}$$

The relative nonlinear error $\left(r_{c}\right)$ of the measurement method based on single capacitance is

(10) $$r_{c}=\frac{|2{\left(\frac{∆d}{d_{0}}\right)}^{2}|}{|2(\frac{∆d}{d_{0}})|}\times 100\%$$

As Equation (7) shows, ε, A, E, and $d_{0}$ can be determined if the material, structure and size of the sensor are determined. The micro-pressure F can be detected according to the output capacitance C. Therefore, we can design the micro-pressure sensor based on parallel plate capacitance according to Equation (7).

**Figure 1:**
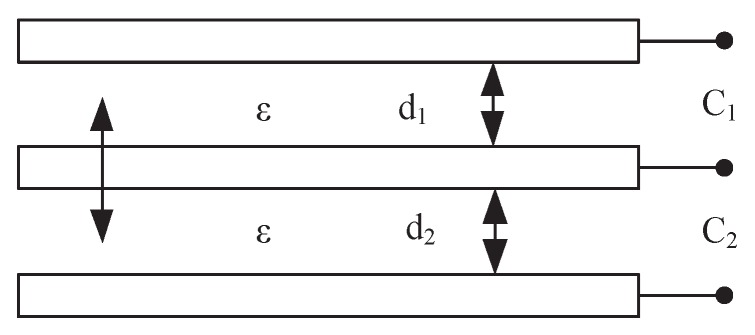
We use a differential parallel plate capacitance structure to compensate the sensitivity, linearity and stability of the capacitance micro-pressure sensor.

In practical applications, due to the high requirement to sensitivity and linearity in the aerospace vehicle micro-pressure measurement, the micro-pressure sensor with the principle of single parallel plate capacitance is easily influenced by electromagnetic attraction, electrostatic attraction, ambient temperature and other factors of interference. It is difficult for the single parallel plate capacitance based sensor to achieve micro-pressure measurement of high sensitivity, high linearity and high stability requirements. Therefore, to compensate sensitivity for the micro-pressure sensor based on the single parallel plate capacitance, we use a differential parallel plate capacitance structure to compensate the sensitivity, linearity and stability of the sensor. The principle of the sensitivity-compensated micro-pressure sensor based on differential parallel plate capacitance is shown in Figure 1. If we set the differential parallel plate capacitance of the sensitivity-compensated micro-pressure sensor in initial position (i.e., $d_{1}=d_{2}=d_{0}$), the upper and lower initial capacitors are equal and the spacing between the plates changes; the plate spacing between $C_{1}$ and $C_{2}$ becomes, $d_{0}+∆d$ and $d_{0}-∆d$ respectively. We can obtain

(11) $$C_{1}=C_{0}\cdot \frac{1}{1+\frac{∆d}{d_{0}}}$$

(12) $$C_{2}=C_{0}\cdot \frac{1}{1-\frac{∆d}{d_{0}}}$$

When $∆d<<d_{0}$, that is, the range is much smaller than the initial distance between the plates, we can obtain the series expansion of the total capacitance change

(13) $$∆C=C_{2}-C_{1}=2\cdot \left[\frac{∆d}{d_{0}}+{\left(\frac{∆d}{d_{0}}\right)}^{3}+{\left(\frac{∆d}{d_{0}}\right)}^{5}+\cdots \right]$$

Omitting the higher term, we can obtain

(14) $$∆C=2C_{0}\frac{∆d}{d_{0}}$$

Substituting Equation (6) into Equation (14), we can obtain

(15) $$∆C=\frac{2\varepsilon F}{d_{0}E}$$

Therefore, the sensitivity of the measurement method ($S^{\prime }$) of the sensitivity-compensated micro-pressure sensor based on the differential plate capacitance is

(16) $$S^{\prime }=\frac{∆C/C_{0}}{F}=\frac{2∆d/d_{0}}{1-{\left(∆d/d_{0}\right)}^{2}}\cdot \frac{1}{F}$$

The relative nonlinear error ($r_{∆C}$) of the sensitivity-compensated micro-pressure sensor based on differential plate capacitance is

(17) $$r_{∆C}=\frac{|2{\left(\frac{∆d}{d_{0}}\right)}^{3}|}{|2(\frac{∆d}{d_{0}})|}\times 100\%$$

As Equation (15) shows, ε, E, and $d_{0}$ can be determined if the material, structure and size of the sensitivity-compensated micro-pressure sensor based on parallel plate capacitance are determined. The micro-pressure (*F*) can be measured according to the output capacitance (∆C). Comparing Equations (9) and (16) with Equations (10) and (17), we found that the sensitivity and linearity of micro-pressure sensor based on differential parallel plate capacitance are significantly higher than that of the micro-pressure sensor based on single parallel plate capacitance. Therefore, we can measure the micro-pressure with sensitivity-compensated micro-pressure sensor based on differential parallel plate capacitance.

### 2.2. Structure Design of the Sensitivity-Compensated Micro-Pressure Flexible Sensor

**Figure 2:**
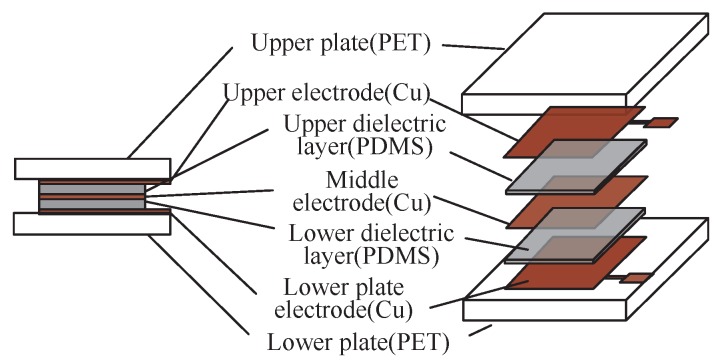
The structure of the sensitivity-compensated micro-pressure flexible sensor.

To realize the sensitivity-compensated micro-pressure flexible sensor presented above, we design a sensor, as shown in Figure 2. The sensor is composed of upper plate, upper plate electrode, upper capacitance dielectric layer, middle electrode, lower dielectric layer, lower plate electrode and lower plate. Two electrodes are on the upper and lower substrate, respectively. An electrode is located between two electrodes separated by two dielectric layers. The upper and lower plates are made of polyethylene terephthalate (PET), because it has excellent physical and mechanical properties, high flexural strength and high Young’s modulus (4000 MPa) to deposit metal electrodes and concentrate the applied micro-pressure [21]. The upper, middle and lower plate electrodes are copper (Cu), because copper has excellent electrical conductivity ($1.75\times 10^{-8}\;\Omega \;\cdot$ m) to produce the plate capacitors. The upper and lower capacitance dielectric layers are composed of polydimethylsiloxane (PDMS) because the sensitivity of the sensor can be effectively improved by using the characteristics of low Young’s modulus (0.55 MPa) with high mass ratio [22] and, for many aeronautical applications, pressure sensors that can be applied on curved surfaces, e.g., on airfoil models, are needed [23]. The working temperature ranges of PDMS, Cu, and PET are −45 °C to 200 °C, up to 1083 °C, and −70 °C to 86 °C, respectively.

Considering the requirement for miniaturization of our sensor, we analyzed the effect of physical dimensions on sensor sensitivity. The initial capacitance value of sensor ($C_{PDMS0}$) is:

(18) $$C_{PDMS0}=\frac{\varepsilon_{0}\varepsilon_{PDMS}A}{d_{0}}$$

where *A* is the overlap area between the plates, $d_{0}$ is the initial distance between the parallel plates, $\varepsilon_{0}$ is dielectric constant of the vacuum and $\varepsilon_{PDMS}$ is the relative dielectric permittivity of the dielectric layer. When the sensor is subjected to a micro-pressure, the distance between the parallel plates decreases, ∆d. The capacitance $C_{PDMS}$ can be written as

(19) $$C_{PDMS}=\frac{\varepsilon_{0}\varepsilon_{PDMS}A}{d_{0}-∆d}=\frac{\varepsilon_{0}\varepsilon_{PDMS}A\left(1+\frac{∆d}{d_{0}}\right)}{d_{0}\left(1-\frac{∆d^{2}}{d_{0}^{2}}\right)}$$

The predicted pressure (*P*) of the thin atmosphere can be converted into force (*F*) by the pressure formula:

(20) F=P×A

By substituting *F* into the definition of Young’s modulus of PDMS, we have

(21) $$E_{PDMS}=\frac{\sigma_{d}}{\varepsilon_{d}}=\frac{F/A}{∆d/d_{0}}$$

By substituting $∆d/d_{0}$ into Equation (19), we have

(22) $$C_{PDMS}=C_{PDMS0}+∆C_{PDMS}=\frac{\varepsilon_{0}\varepsilon_{PDMS}E_{PDMS}A^{2}}{d_{0}\left(E_{PDMS}A-F\right)}$$

The sensitivity of the sensor can be written as

(23) $$S=\frac{∆C_{PDMS}/C_{PDMS0}}{F}=\frac{∆d}{d_{0}-∆d}\cdot \frac{1}{F}$$

When $∆d<<d_{0}$, the range is much smaller than the initial distance between the plates. Thus,

(24) $$C_{PDMS}\approx \frac{\varepsilon_{0}\varepsilon_{PDMS}}{E_{PDMS}d_{0}}F+\frac{\varepsilon_{0}\varepsilon_{PDMS}A}{d_{0}}$$

Similarly, the sensitivity can be written as

(25) $$S\approx \frac{∆C_{PDMS}/C_{PDMS0}}{F}=\frac{∆d}{d_{0}}\cdot \frac{1}{F}$$

The output capacitance of the sensor is related to the Young’s modulus of the dielectric layer ($E_{PDMS}$), the thickness of the dielectric layer ($d_{0}$) and the overlap area of the dielectric layer (*A*). The lower the Young’s modulus ($E_{PDMS}$) gets, the higher sensitivity of the sensor (*S*) will be. The smaller the thickness of the dielectric layer ($d_{0}$) is, the smaller the output capacitance of the sensor will be, and, under the same Young’s modulus, the higher the sensitivity (*S*) will be. The smaller the dielectric layer area (*A*) is, the smaller the changes of output capacitance will be. Therefore, considering the requirements of miniaturization, flexibility and the application scenarios of the sensor, we designed the overlapping area (*A*), the thickness ($d_{0}$) of the dielectric layer, and the PDMS mixing ratio to be 10 mm × 10 mm, 500 µm, and 20:1, respectively.

**Figure 3:**
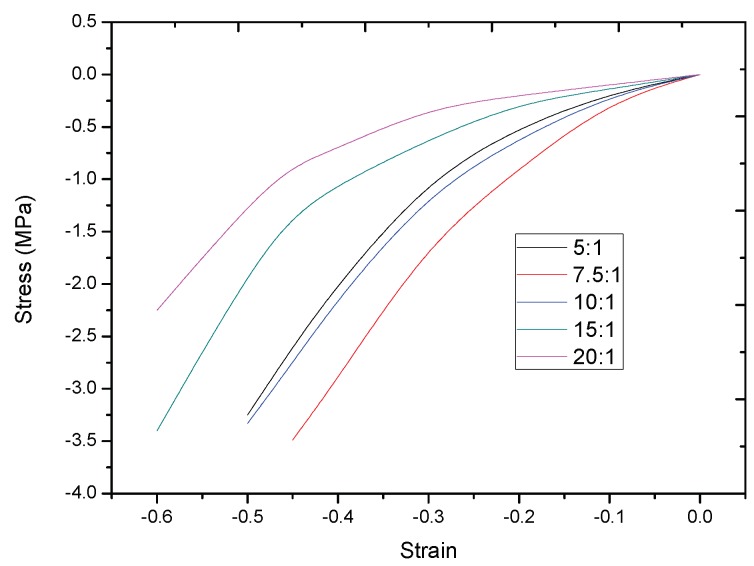
Stress–strain relation of uniaxial tensile compression test.

Compared with other flexible materials (PET, PI, PC, PMMA, etc.), we chose PDMS (Sylgard 184, Dow Corning Co., Midland, MI, USA) as the dielectric layer because PDMS has a low Young’s modulus. The specific Young’s modulus of PDMS is related with the ratio of the base and curing. To chose a proper ratio between the base and curing of PDMS, we tested the stress–strain relation of uniaxial tensile compression of PDMS with different base material and curing material ratio (see Figure 3). From the result, we obtained that the modulus will be smaller if the ratio is bigger. In this work, we chose a small ratio of 20:1 to make the sensor more sensitive, and the Young’s modulus is 0.55 MPa.

Because the sensor will be applied in a narrow space of aerospace vehicle and suffer a micro-pressure in near space, the size of the sensor should meet the requirement of miniaturization and flexibility. Compared with decreasing the thickness of the dielectric layer ($d_{0}$), it is more difficult to measure the tiny output capacitance changes when decreasing the dielectric layer area (*A*). However, we cannot satisfy the miniaturization requirement of the sensor if the area of the dielectric layer (*A*) is too large. Comprehensively considering the requirement of miniaturization and high sensitivity, we selected the dielectric layer area (*A*) and the thickness ($d_{0}$) with the size of 1 cm × 1 cm and 500 µm, respectively.

We chose polyethylene terephthalate (PET) as the upper and lower substrates because PET has a high Young’s modulus, and can reduce the absorption of the micro-pressure applied to the sensor. The upper and lower substrates were designed for data measurement and performance analysis with the size of 1.5 cm × 1.5 cm and 0.2 mm, respectively, because of the requirement of the follow-up measurements with lead wire [24]. The electrodes were prepared by MEMS preparation process, and the photolithography precision is up to 0.3 µm. We used a high-precision cutter to cut PET with accuracy up to 0.5 mm.

Therefore, we used the 20 : 1 mass ratio of PDMS as the dielectric layer of the micro-pressure sensor, which has a low Young’s modulus (*E*) of 0.55 × 10^6^ F/m^2^. The upper and lower capacitance dielectric has an area (*A*) of 1.0 cm × 1.0 cm and thickness ($d_{0}$) of 500 µm. The area of electrode is same with the dielectric layer 1.0 cm × 1.0 cm and the thickness of the electrode is 100 nm cite. The upper and lower substrates were designed with the size of 1.5 cm × 1.5 cm and 0.2 mm, respectively, to facilitate lead wire.

## 3. Materials

**Figure 4:**
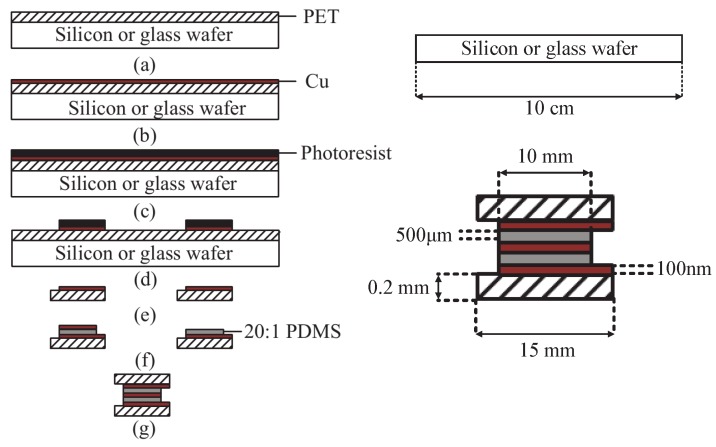
The fabrication process of the sensitivity-compensated micro-pressure flexible sensor device.

**Figure 5:**
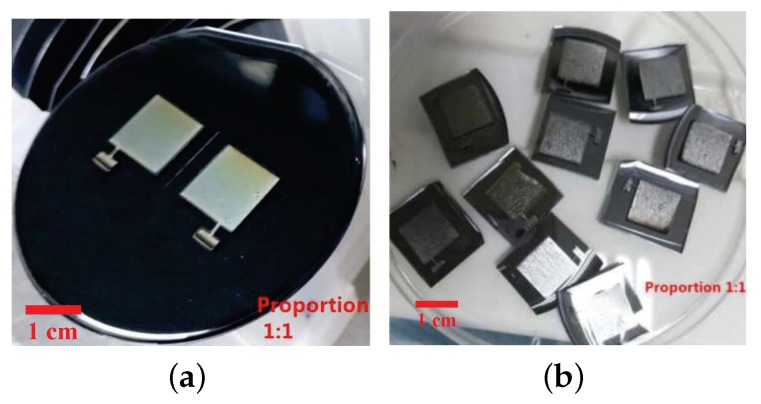
(**a**) The photographs of the upper and lower substrates that pattern before cutting. (**b**) The photographs of the upper and lower substrates that pattern after cutting.

**Figure 6:**
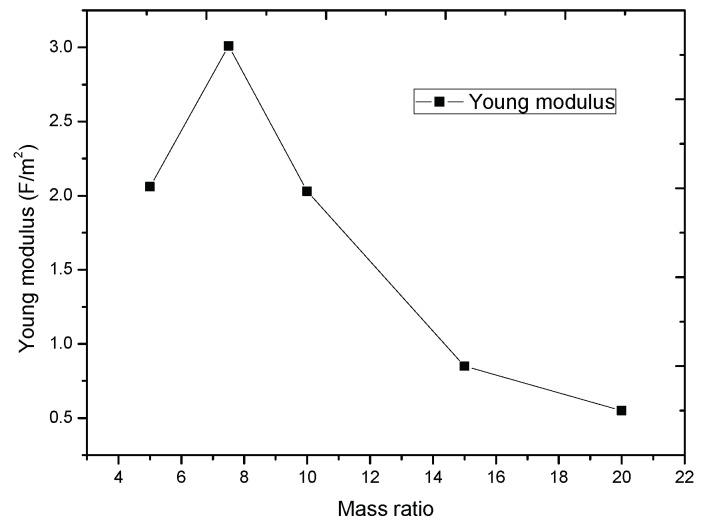
The Young’s modulus of different mass ratio of PDMS.

**Figure 7:**
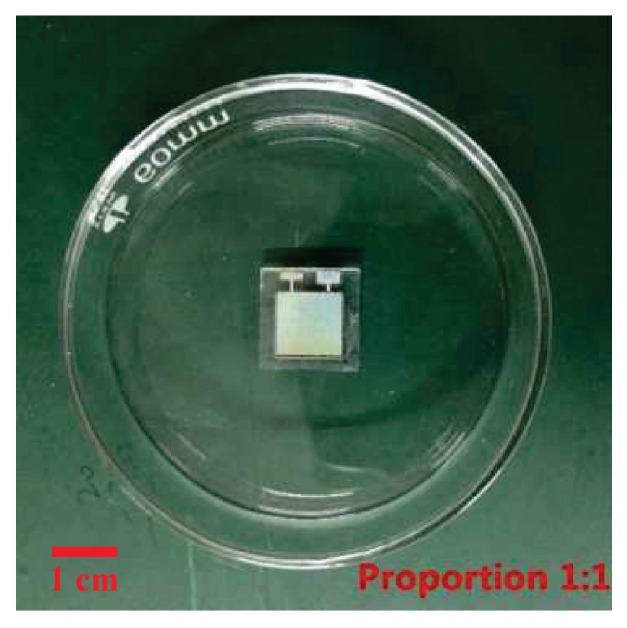
The photograph of the fabricated sensor.

To implement the sensitivity-compensated micro-pressure flexible sensor presented above, sample devices were fabricated. The fabrication process is shown in Figure 4. The specific steps of the fabrication process were: (1) We selected PET (diameter: 10 cm) to manufacture the upper and lower substrates because of its large Young’s modulus and its ability to concentrate the micro-pressure of the surface.The PET (Honuo Plastic Insulation Materials Co. LTD, China) was taped on a silicon or glass wafer. The tape should cover all sides of the PET [25]. Then, we used magnetron sputtering technology to sputter Cu on the PET surface with a thickness of 100 nm. (2) We spin-coated the surface with a layer of photoresist (AZ-1500, Shipely) and depicted the design of the electrode pattern on the upper plate and lower substrate after photolithography technique, exposure, development resisting removal, and cutting technologies. The photographs of the fabricated upper and lower substrates are shown in Figure 5. (3) The stress-train of uniaxial tensile and Young’s Modulus of PDMS will vary according to the mass radio of based material and curing material (See Figure 3 and Figure 6). In this work, The PDMS dielectric layer has a thickness of 500 µm and spin-coated by a Spin Coater (KW-4B, Institute of Microelectronics of the Chinese Academy of Sciences) for 10 s at 500 rpm. (4) We used magnetron sputtering technology to sputter Cu on the lower dielectric layer’s surface with a thickness of 100 nm. Then, we used heating semi-cured packaging technology [26] to fit the upper and lower plate. The PDMS was first pre-heated for a short period. In this state, the PDMS has a solid form and is adhesive and can adhere to other materials. The photograph of the packaged and integrated sensor is shown in Figure 7.

## 4. Results and Discussions

### 4.1. Experiment Setup

**Figure 8:**
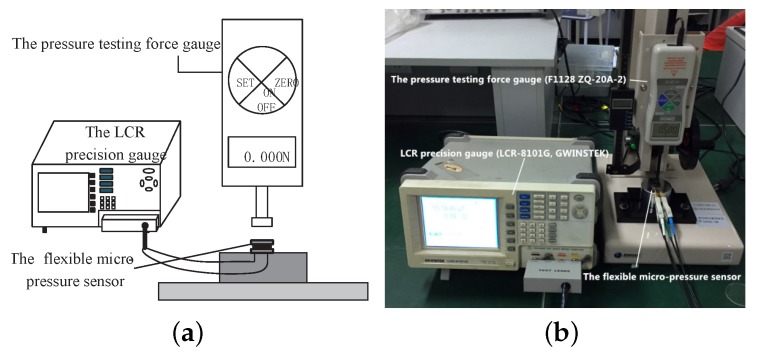
The sensor was tested by a pressure simulation experiment system. (**a**) The diagram of the experiment setup. (**b**) The photograph of the experiment setup.

To test the performance of the sensor fabricated above, an experiment was assembled, as shown in Figure 8. The sensor was placed on a measuring platform. A pressure testing force gauge simulated and applied micro-force on the sensor. A capacitance measuring instrument was connected to the sensor and recorded the output capacitance. In this work, we applied a range of 0–6 kPa micro-force by the pressure testing force gauge (F1128 ZQ-20A-2, ZHIQU Precision Instruments). Then, we used the LCR precision gauge (LCR-8101G, GWINSTEK, Taiwan) to measure the output capacitance of the sensor. The voltage and frequency of the LCR meter are 1 V and 1 MHz, respectively.

### 4.2. Static Characteristic

**Figure 9:**
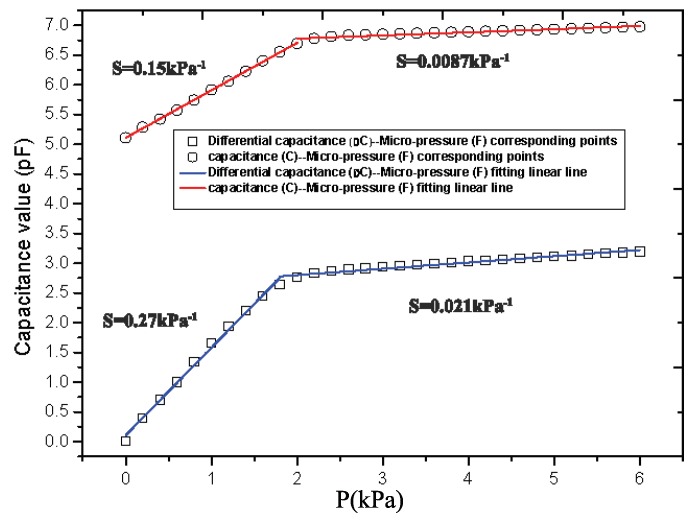
The static characteristic of the sensor without sensitivity compensation and with sensitivity compensation structure.

The static characteristic of the sensor without sensitivity compensation and with sensitivity compensation structure are shown in Figure 9. Both the output capacitance curve with sensitivity compensated structure (∆C) and output capacitance curve without sensitivity compensated structure (*C*) under different pressure can be approximated in two straight lines (as shown in Equations (26) and (27)).

(26) $$∆C=\left\{\begin{array}{ccc} 1.407P+0.15 & & P<2\;\mathrm{kPa}\\ 0.096P+2.64 & & 2.2\;\mathrm{kPa}<P<6\;\mathrm{kPa} \end{array}\right.$$

(27) $$C=\left\{\begin{array}{ccc} 0.788P+5.12 & & P<2\;\mathrm{kPa}\\ 0.043P+6.72 & & 2.2\;\mathrm{kPa}<P<6\;\mathrm{kPa} \end{array}\right.$$

From the results in Figure 9, we found that the static characteristic curve’s slope changes at 2 kPa. This slope change appears because the Young’s modulus of PDMS becomes larger after PDMS deforms to a certain extent. Then, the varying Young’s modulus resulted in a different slope of the output curve with the micro-pressure change before and after the 2 kPa [27,28]. From Equation (17), we concluded that the change of the two types of flexible micro-pressure sensor are both nonlinear, but could be approximated as two linear curve in the ranges of 0–2 kPa and 2–6 kPa. The dielectric layer of the sensor is PDMS, which has good compressibility. Its Young’s modulus is 0.55 MPa in the ratio of 20:1. When a pressure is applied to the surface of the sensor, the upper and lower dielectric layers are compressed due to the pressure, the distance between the capacitance plates of the sensor decreases, and the capacitance of the sensor increases accordingly. Due to the hyperelastic property of PDMS, the pressure increases nonlinearly with the compression of PDMS. Therefore, the sensitivity decreased obviously after 1.6 kPa.

### 4.3. Sensitivity

According to the experiment results, we obtained that the initial value of the single capacitance structure micro-pressure sensor is 5.111 pF and the capacitance value (*C*) is 6.809 pF at 2 kPa and 6.997 pF at 6 kPa. The initial differential value of the differential capacitance structure micro-pressure sensor device is 5.152 pF and the differential capacitance value (∆C) is 2.765 pF at 2 kPa and 3.196 pF at 6 kPa. Therefore, we can determine the full-scale output, the full-scale input and the initial value of these two type sensor devices, respectively. According to Equation (9), we can obtain the sensitivities of these two sensor devices are

(28) $$S_{∆C1}=\frac{\left(2.765-0.011\right)/5.152}{2}=0.27\;{\mathrm{kPa}}^{-1}$$

(29) $$S_{∆C2}=\frac{\left(3.196-2.765\right)/5.152}{4}=0.021\;{\mathrm{kPa}}^{-1}$$

(30) $$S_{C1}=\frac{\left(6.809-5.111\right)/5.111}{2}=0.15\;{\mathrm{kPa}}^{-1}$$

(31) $$S_{C2}=\frac{\left(6.977-6.809\right)/5.111}{4}=0.0087\;{\mathrm{kPa}}^{-1}$$

The sensitivity of the differential capacitance structure micro-pressure sensor is 0.27 kPa^−1^ at 0–2 kPa and 0.021 kPa^−1^ at 2–6 kPa. The sensitivity of the single capacitance structure micro-pressure sensor is 0.15 kPa^−1^ at 0–2 kPa and 0.0087 kPa^−1^ at 2–6 kPa. The sensitivities of the differential capacitance structure micro-pressure sensor device within 0–2 kPa and 2–6 kPa are higher by 80% and 141.38%, respectively, than that of the single capacitance structure micro-pressure sensor device. Therefore, the sensitivity of the micro-pressure sensor device is obviously improved by the differential capacitance structure.

### 4.4. Linearity

Linearity ($\delta_{L}$) is a symbol about the degree of deviation between calibration curve and fitting curve. Linearity describes the percentage of the maximum deviation ($∆Y_{\max}$) between the sensor calibration curve from the fitted line and the full-scale output (Y). According to the experiment results, we obtained that the linearities of the two different structures of micro-pressure sensor devices are

(32) $$\delta_{∆C_{L1}}=\frac{∆Y_{\max}}{Y}\times 100\%=\frac{2.6785-2.6392}{2.9599-0.1459}\times 100\%=1.39\%$$

(33) $$\delta_{∆C_{L2}}=\frac{∆Y_{\max}}{Y}\times 100\%=\frac{3.1876-3.1773}{3.2055-2.8471}\times 100\%=2.88\%$$

(34) $$\delta_{C_{L1}}=\frac{∆Y_{\max}}{Y}\times 100\%=\frac{6.8397-6.7788}{6.8397-5.1202}\times 100\%=3.54\%$$

(35) $$\delta_{C_{L2}}=\frac{∆Y_{\max}}{Y}\times 100\%=\frac{6.8238-6.8309}{6.9808-6.8053}\times 100\%=4.07\%$$

The linearity of the differential capacitance structure micro-pressure sensor is 1.39% at 0–2 kPa and 2.88% at 2–6 kPa. The linearity of the single capacitance structure micro-pressure sensor is 3.54% at 0–2 kPa and 4.07% at 2–6 kPa. Therefore, the linearity of the differential capacitance micro-pressure device within 0–2 kPa and 2–6 kPa are higher by 51.7% and 13.1%, respectively, than that of the single capacitance structure micro-pressure sensor. The linearity of the micro-pressure device after sensitivity compensation is obviously improved.

### 4.5. Hysteresis

**Figure 10:**
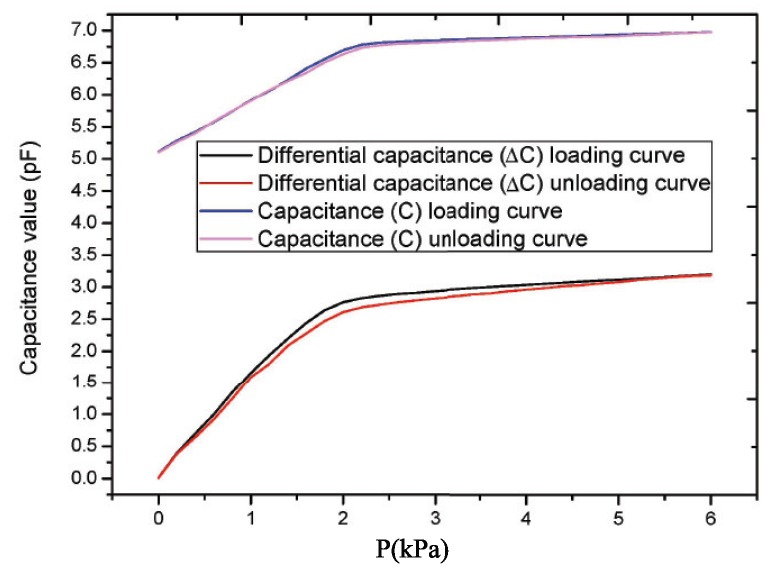
Hysteresis curves of differential capacitance and single capacitance structure flexible micro-pressure sensor devices.

Hysteresis ($\delta_{H}$) is the maximum difference between the loading and unloading stroke calibration curves when the sensor is calibrated for the full measurement range under the same operating conditions [7]. It is expressed numerically by the percentage of full scale output (Y) with the maximum difference ($∆H_{\max}$). The positive and negative stroke curves of two different structures of micro-pressure sensor devices are shown in Figure 10. The hystereses of the two different structures of micro-pressure sensor devices can be given by

(36) $$\delta_{H}=\frac{∆H_{\max}}{Y}\times 100\%=\frac{2.7651-2.6074}{3.1958-0.0118}\times 100\%=4.95\%$$

(37) $$\delta_{H}=\frac{∆H_{\max}}{Y}\times 100\%=\frac{6.77884-6.73181}{6.97749-5.11131}\times 100\%=2.52\%$$

The hystereses of the differential and single capacitance flexible micro-pressure devices are 4.95% and 2.52%, respectively. The hysteresis of the single capacitance structure flexible micro-pressure device is better than that of the differential capacitance structure device. The reasons are: (1) when measuring the differential capacitance, the tension pressure tester produces different parasitic capacitance interference after loading and unloading the pressure. (2) Because the experimental device can only measure the single capacitance value, the environmental influence factors are introduced into the measurement of capacitance, such as redundant edge effects, electrostatic attraction, parasitic capacitance, environmental measurement error and other factors, which reduce the hysteresis performance of differential capacitance measurement devices.

### 4.6. Repeatability

**Figure 11:**
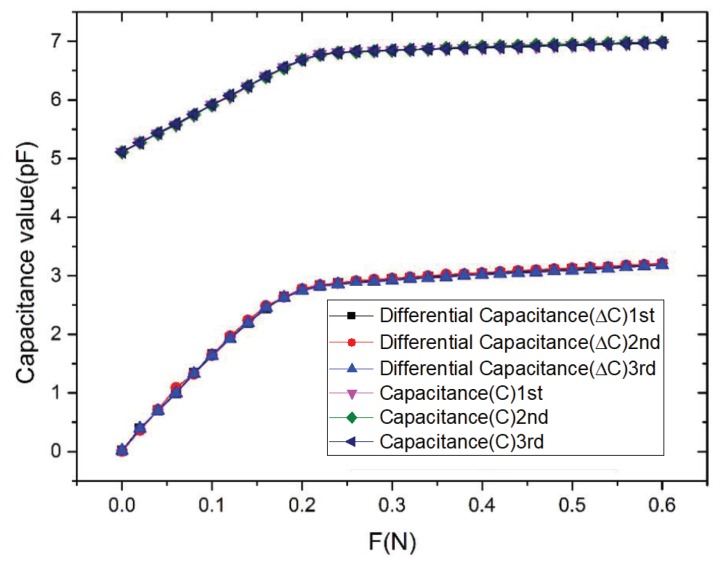
The repeatability curve of the sensor.

The repeatability of sensor is important to the practical application of the sensor. Figure 11 presents the curves of the repeatability experiment. We could obtain the repeatability of the differential capacitance sensor as

(38) $$\sigma =\sqrt{\frac{\sum_{i=1}^{n}{\left(F_{i}-\bar{F}\right)}^{2}}{n-1}}=0.02527$$

(39) $$\delta_{∆C}=\frac{3\cdot \bar{\sigma }}{F_{FS}}\cdot 100\%=2.38\%$$

where $\bar{\sigma }$ is the mean standard deviation of the sensor; $F_{FS}$ is the full measurement range of the sensor; *i* is the index of the measurement point; $F_{i}$ is the corresponding measurement; and $\bar{F}$ is the average value of the measurement points. By substituting $\bar{\sigma }$ into Equation (38), we obtained that the repeatability of the sensor is 2.38%. Similarly, according to Figure 11, the repeatability of the signal capacitance sensor is 2.19%. The result shows that the repeatability of the differential capacitance sensor is larger than the single capacitance sensor. We think the decrease of the repeatability of the differential capacitance sensor is due to the coupling capacitance and stray capacitance in the measurement of LCR. It can be seen from the above results that, compared with the single capacitance sensor, the differential capacitance sensor can increase the sensitivity (from 0.27%/mN to 0.15%/mN in the range of 0–200 mN, and from 0.021%/mN to 0.0087%/mN in the range of 220–200 mN) and the linearity (from 3.54% to 1.39% in the range of 0–200 mN, and from 4.07% to 2.88% in the range of 220–200 mN), but decrease the hysteresis (from 2.52% to 4.95%) and the reproducibility (from 2.19% to 2.38%).

### 4.7. Influence of Flexibility

**Figure 12:**
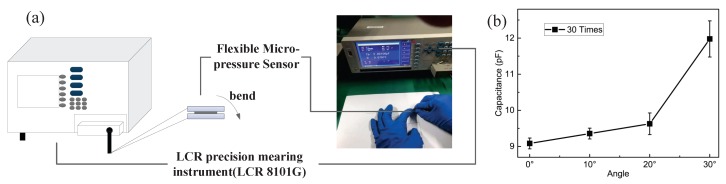
The output capacitance is influenced by the angle of flexibility: (**a**) flexibility experiment; and (**b**) the results of the influence of flexibility.

To test the influence of flexibility to the sensor, we carried out the experiment shown in Figure 12a. The sensor was bent at angles from 0° to 30°, 30 times. The output capacitance of the sensor was measured by LCR precision measuring instrument (LCR 8101G), as shown in Figure 12b. From the results in Figure 12b, we obtained that the output capacitance varied by 12 pF when the sensor was bent to 30°, which is 1 pF per degree. We also obtained that the variety of the output capacitance changed linearly with the bending degree. That means the influence of the flexibility of the sensor can be compensated easily.

### 4.8. Temperature Stability

**Figure 13:**
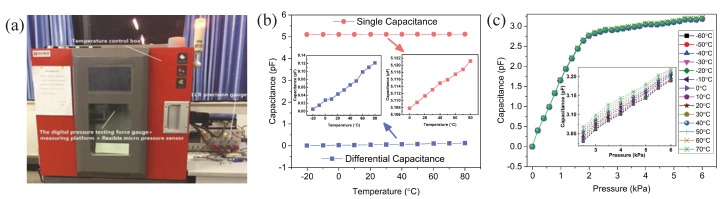
(**a**) Photograph of the experiment; (**b**) the capacitance of the sensor under different temperature; and (**c**) the capacitance of the sensor under different temperature and force.

Temperature stability is the changes of the sensor at different temperature. To investigate the temperature stability of our sensor, we placed the digital force gauge and the sensor measurement platform in the temperature control box, leading the sensor connecting wire out of the temperature control box. Then, we used LCR precision gauge to measure the sensor output value under different temperature and different micro-pressure, as shown in Figure 13a. We measured the output of the sensor in the temperature range of −60 °C to 70 °C with a step of 10 °C. The output of the sensor with no loading pressure and with loading pressure are shown in the Figure 13b,c. The temperature stability errors can be written as

(40) $$\delta_{T\max}=\frac{{∆}_{\max}}{C}\times 100\%$$

where $\delta_{T\max}$ is the maximum value of the temperature stability relative error at every 10 °C, ${∆}_{\max}$ is the maximum value of the temperature stability absolute error at every 10 °C, and *C* is the output of the sensor. According to the calculation of Equation (40), the maximum temperature stability relative error of the sensor from −60 °C to 70 °C is 0.148%. The cause of generating such an error is PDMS has a small expansion coefficient, which leads the dielectric layer forms different deformation at different temperatures. This temperature stability relative error value means the flexible micro-pressure static input and output characteristics is very stable and almost unaffected by changes in temperature from −60 °C to 70 °C.

## 5. Conclusions

In this paper, a sensitivity-compensated micro-pressure flexible sensor for flight vehicles is designed based on plate capacitance and hyper-elastic material. We propose the principle, structure design and fabrication of the sensitivity-compensated micro-pressure flexible sensor. We carried out experiments to obtain the static characteristic curve between micro-pressure and the output capacitance of the sensor devices, and investigated the relationship between sensitivity and geometric parameters. We also compared the performance of the flexible sensor before and after sensitivity compensation. The results show that the sensor can measure a range of 0–2 kPa and 2–6 kPa with a sensitivity of 0.27 kPa^−1^ and 0.021 kPa^−1^, which are 80% and 141.38% higher than the sensor before compensation; a linearity of 1.39% and 2.88%, which are 51.7% and 13.1% higher than the sensor before compensation; and a hysteresis and repeatability of 4.95% and 2.38%, respectively. The measuring method has potential application in flight vehicles to measure the micro-pressure with high sensitivity, high linearity and high stability. Future works will focus on improving the sensitivity, stability, and adaptability. First, to modify the dielectric silicone, the dielectric with lower Young’s modulus or high permittivity may increase the sensitivity. Second, reducing or eliminating the parasitic and stray capacitance in the capacitive sensor should improve the stability. Third, encapsulating the sensor may enhance the adaptability in flight vehicle environment conditions, such as vacuum and radiation.
